# Basic Concepts in Genetics and Pharmacogenomics for Pharmacists

**DOI:** 10.1177/1177392819886875

**Published:** 2019-12-03

**Authors:** Kathleen B Orrico

**Affiliations:** 1 School of Pharmacy, University of California, San Francisco, San Francisco, CA, USA; 2 Center for Clinical Research, Stanford University School of Medicine, Stanford, CA, USA

**Keywords:** pharmacogenomics, pharmacogenetics, personalized medicine, pharmacogenomics knowledge base

## Abstract

This basic review of genetic principles will aid pharmacists in preparing for their eventual role of translating gene-drug associations into clinical practice. Genes, which are stretches of deoxyribonucleic acid (DNA) contained on the 23 pairs of human chromosomes, determine the size and shape of every protein a living organism builds. Variation in pharmacogenes which encode for proteins central to drug action and toxicity serves as the basis of pharmacogenomics (PGx). Important online resources such as PharmGKB.org, cpicpgx.org, and PharmVar.org provide the clinician with curated and summarized PGx associations and clinical guidelines. As genetic testing becomes increasingly affordable and accessible, the time is now for pharmacists to embrace PGx-guided medication selection and dosing to personalize and improve the safety and efficacy of drug therapy.

We are at a pivotal moment in medical science and pharmacy practice where specific variations in the genetic code are being associated with differences in drug response, an individual’s propensity for developing certain drug side effects, and variation in the rate and extent of drug metabolism. Termed pharmacogenomics (PGx), this new discipline is the study of the interplay between the human genome and the science of pharmacology.^1^ The curriculums of most pharmacy schools have only recently included coursework in genetics and PGx. Pharmacists educated in the past may have had little exposure to the genetic principles underlying PGx. The intent of this review is to present basic concepts in genetics and genetic variation to provide a foundation for understanding important and highly evidenced gene-drug associations. Whenever possible, the examples used will include medications where genetic testing is recommended by the US Food and Drug Administration (FDA) labeling or expert clinical practice guidelines. Several reliable, online PGx references will also be presented. As pharmacists and scientists, the time is now to embrace these ensuing advancements in drug therapy and prepare ourselves to translate gene-drug associations into clinical practice.

Pharmacogenomics was born out of the findings from the Human Genome Project (HGP; www.genome.gov).^2^ Launched in 1990, the HGP was an international effort to identify and understand the structure of every gene human beings possess. The HGP will no doubt be viewed as the greatest scientific advancement of our lifetime and will have a profound impact on medical science and the practice of pharmacy. The main mission of the project was to crack the human genetic code by combining the power of scientists from universities and research centers in the United States, the United Kingdom, France, Germany, Japan, and China. This worldwide endeavor stands as a testimony to the greater good that can be achieved when researchers work together to accomplish a common goal. The sequencing or mapping of the 2.91 billion base pairs that spell out the genetic blueprint within the molecule called deoxyribonucleic acid (DNA) was completed years ahead of schedule in April of 2003. The entire code is known as the human genome and is housed in the nucleus of nearly every human cell. Research to discover how variation and expression of this code influence human health is just beginning.^3^ The HGP has given rise to truly personalized medicine, which relates markers or patterns in an individual’s DNA sequence to the causation and treatment of disease.

Pharmacists are the ideal practitioners to implement PGx-guided drug therapy and to translate gene-drug associations into clinical practice. Pharmacists will be needed to personalize the selection and dosing of medications and explain PGx risk information to both patients and providers. The intent of this review is to open a window into the future of drug therapy by presenting the basic terminology and concepts of PGx and provide educational resources that will help you remain up to date with these emerging changes in medical science and medication management.

## DNA, Chromosomes, and Genes

A foundational concept to keep in mind is that genes, which are stretches of DNA, determine the composition, size, and shape of every protein a living organism builds. One gene may hold the “recipe” for one or hundreds of different proteins. An effort to identify every protein human beings produce called the Human Proteome Project is also underway and once completed humans are estimated to make approximately 250 000 to 1 million different proteins.^4^ As enzymes, transporters, drug receptor, binding sites, cell structural components, and peptide hormones, protein molecules are central to nearly every biochemical and pharmacological reaction.

Proteins are unique molecules in that their function is greatly affected by their conformational shape. Variation in the amino acid composition of a protein, which is translated from a gene(s), can influence molecular folding, and therefore the biological activity of the polypeptide molecule produced. For example, the *CFTR* gene “encodes” for a protein structure called the Cystic Fibrosis Transmembrane Conductance Regulator (CFTR), which serves as a channel for chloride ions across exocrine cell membranes mainly in lung and pancreatic duct epithelium. Cystic fibrosis is caused by variations or mutations in the *CFTR* gene, which result in CFTR proteins that differ in structure and possess limited to no ability to transport chloride and other ions. This changes the electrolyte composition and viscosity of exocrine secretions leading to cystic fibrosis disease sequelae. The most common *CFTR* gene variation is caused by the deletion of 1 phenylalanine amino acid in the CFTR protein produced. This singular change in CFTR structure allows it to be sequestered and destroyed by regulatory factors before it can be placed within the cell membrane.

**Figure 1.**
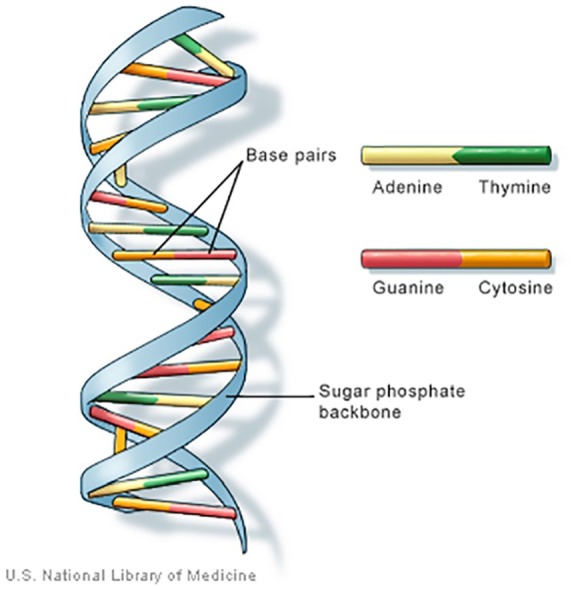
Double helix structure of deoxyribonucleic acid (DNA). Source: Genetics Home Reference.^5^

The genome or complete set of genetic instructions for all cellular organisms such as plants, animals, and human beings is spelled out in the structure of DNA and serves as the molecular basis of inheritance.^5^ The DNA molecule is a large double-stranded polymer of nucleotide base units that resembles a twisted ladder and is often referred to as a double helix (Figure 1). The side rails or backbone of the ladder are composed of repeating units of the 5-carbon sugar deoxyribose linked by an acidic phosphate group. The rungs of the ladder are formed from the hydrogen bonding of 2 of 4 different nitrogenous bases and serve as the connections between the 2 nucleotide strands. Much as binary code is to computer language where every instruction is written as a series of 0s and 1s, it is the sequence or pattern of these 4 nitrogenous bases that ultimately spells out the directions to make every protein. The 4 bases in DNA’s alphabet include the double-ring purines adenine (A) and guanine (G) and the single-ring pyrimidines cytosine (C) and thymine (T); 1 purine and 1 pyrimidine form the connecting or complementary base pairs in a very specific manner. Adenine always pairs with thymine (AT) using 2 hydrogen bonds, and guanine always pairs with cytosine (GC) using 3 hydrogen bonds. Therefore, it is only necessary to determine the order of bases in 1 strand of the DNA molecule to deduce the sequence of the complementary strand.

Within the nucleus of every human cell except red blood cells and platelets, DNA is arranged into structures known as chromosomes. A small amount of DNA, inherited through the maternal line only, exists in the mitochondria of cells and is known as mitochondrial DNA (mtDNA).

If stretched end to end as a continuous strand, the DNA contained in 1 cell nucleus called nuclear DNA (nDNA) measures over 6 feet in length and contains all of the approximately 3 billion base pairs that make up the human genome. Before mitosis or cell division begins, nDNA tightly coils around histone proteins to form structures called chromosomes. Much more than inert spools, the function of histone proteins has only begun to be appreciated. Through winding and unwinding of the DNA strand in response to intracellular signaling, histones orchestrate gene exposure to the cellular environment and play a role in gene expression.

**Figure 2.**
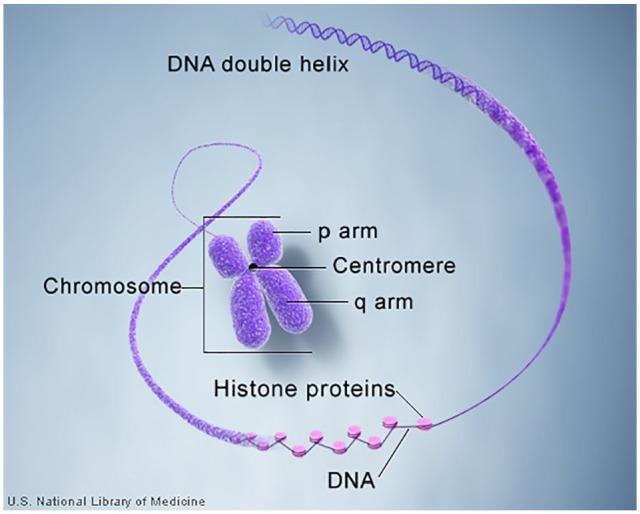
The structure of a human chromosome. DNA indicates deoxyribonucleic acid. Source: Genetics Home Reference.^7^

Chromosomes are not static structures but rather active arrangements of specific sections of the nDNA strand that comprise the whole genome. The size of human chromosomes ranges from 50 000 000 to 300 000 000 base pairs and can contain hundreds to thousands of genes.^6^ Chromosome 1, for example, which is the largest, contains approximately 2100 protein-coding genes, while the Y chromosome contains the least number or 60 functional genes. Chromosomes are typically depicted as in Figure 2, when they are most visible. This arrangement occurs immediately before cell division and after the DNA has been replicated forming 2 sister chromatid strands held in contact by a centromere. Within the nucleus of every cell, except sperm and egg cells, humans have a total of 46 chromosomes existing as 23 pairs composed of 1 chromosome contributed from the mother and 1 from the father; 22 of these pairs are called autosomes and are identified by number from the largest in size (chromosome 1) to the smallest (chromosomes 22). The 23rd or last pair are the non-identical sex chromosomes. Named the X and Y chromosomes, they determine the sex of the offspring.

At the ends or tips of the DNA strands that form chromosomes are repeating sequences of base pairs called telomeres.^8^ Rather like caps or aglets at the end of shoelaces, telomeres protect the chromosome from breakdown by providing spare parts for the ongoing process of DNA repair that occurs during replication. An association exists between the length of telomeres and the life span of a cell, and thus the ultimate age of a living organism.

## Genes

A gene is a specific section of the DNA base pair sequence located on the chromosome, which acts as a recipe or code that is transcribed and then translated into the amino acid structure of a protein. Of the estimated 20 000 protein-coding genes in the human genome, each makes an average of 3 proteins.^7^ The gene’s DNA sequence is divided into regions called exons and introns. During transcription, messenger ribonucleic acid (mRNA) copies or transcribes the code, and delivers it to the ribosome for translation to a protein. It is the exons or protein-coding portions of the sequence that ultimately determine which amino acids comprise a given protein. The intron sections perform regulatory functions and are spliced out. The size of a gene is expressed by the number of nitrogenous base pairs it contains and can range from a few hundred to over 2 million.

Many genes are named for the proteins they encode and are given a symbol. For example, the Cytochrome P450 (CYP) 2D6 enzyme and gene share the same name, and the gene is assigned the symbol *CYP2D6*. The gene symbol *ACE* designates the angiotensin I converting enzyme gene. The Human Genome Organisation (HUGO) Gene Nomenclature Committee (genenames.org) establishes an official name and symbol for each gene; however, be aware that genes may be referred to by multiple names in the literature.^9^

Genes are the basic units for the inheritance of traits. Genes that encode for proteins involved in drug action, toxicity, or metabolism are often referred to as pharmacogenes. The term *phenotype* is used to designate the type or classification of a visible trait or characteristic resulting from gene expression such as eye color, height, or the rate of cytochrome p450 (CYP P450) metabolism. At the time of this writing, 66 “Very Important Pharmacogenes” (VIPs) are listed by the premier curators of PGx information *The Pharmacogenomic Knowledge Base* (PharmGKB).^10^ Chief among these are genes for specific CYP P450 enzymes with known associations between variation in their genetic recipe and the phenotype or degree of enzyme activity produced by the resulting protein.

Human beings are diploid organisms, meaning we have 2 versions of every gene, 1 inherited from each parent. The process of meiosis determines which 1 of the 2 genes each parent possesses gets inherited by the offspring and is the basis for the Mendelian Law of Segregation. When egg and sperm combine, the now-fertilized egg contains the entire genome of the offspring which in turn gets passed to future cells at every cell division. The term *genotype* refers to the specific combination of the 2 genes (alleles) an individual inherits.^11^ Alleles are forms or variants of the same gene with small differences in their DNA base sequence. Differences in the DNA base sequence can ultimately lead to variation in the structure of the encoded protein or the amount produced. Some genes are more likely than others to be subject to variation, and the term *polymorphism* (multiple forms) is generally used to discuss genes with multiple alleles. Most of the genes located on autosomal chromosomes have biallelic expression, meaning that both copies express the encoded protein. Other factors involved in gene expression can influence when or whether a protein will be made and in what amount. Understanding the implications of variation in pharmacogenes is the heart and soul of PGx and allows us to individualize drug therapy.

As a species, human beings are nearly genetically homogeneous and share over 99.9% of our entire genome with each other. It is this less than 0.1% variation that makes each of us unique. A set or group of gene alleles typically located on the same chromosome and inherited together is referred to as a haplotype and may indicate a common line of descent in individuals who share one. The allele or version of a gene that produces a trait shared by a sizable group of individuals in a population is often referred to as the non-mutated or wild-type allele.^11^ The wild-type variant(s) is often noted using star (*) nomenclature and written as the gene symbol followed by an asterisk and number 1 (CYP2D6*1). It is possible to have more than 1 wild-type allele typically noted by a letter following *1.

A person’s pharmacogenes can differ in multiple ways. Genetic variation that results from differences in the DNA structure of a gene or regulatory portions of the genome can be caused by deletions, insertions, duplications, inversions, or substitutions of nitrogenous bases within the usual sequence, thus changing the genetic code and possibly the proteins produced. A single-nucleotide polymorphism (SNP, “snip”) occurs when 1 nitrogenous base, along with its paired complement, is substituted within the nucleotide sequence. Single-nucleotide polymorphisms can be conceptualized as misspellings or “typos” in the usual order of bases. A SNP can become consequential if it occurs within a gene or regulatory region, and alters the function of the protein produced or disrupts the usual recipe so that no protein is produced at all. For example, an allele of the *CYP2C19* gene named CYP2C19*2A (“star 2A”) results from a substitution of an adenine instead of a guanine at exon position 681 (noted as 681G>A) and results in a non-functioning enzyme.^12^ Several drugs, including the anti-platelet agent clopidogrel, require *CYP2C19* for bioactivation to an active metabolite to be fully effective. People receiving clopidogrel, whose genotype includes 1 or 2 no function alleles such as CYP2C19*2A, are classified as Poor Metabolizers (PMs) of the drug. The FDA-approved label for clopidogrel recommends that people who are PMs consider use of an alternative platelet P2Y12 inhibitor to avoid poor outcomes.^13^

Copy number variation (CNV) occurs when sections of the usual DNA sequence either repeat multiple times or are deleted and do not occur at all.^14^ Copy number variations become significant when they encompass a regulatory or coding section of a gene. When CNV leads to greater than 2 copies of a gene, the encoded protein may be produced in greater amounts and enzymatic activity increased. A CNV, that results in multiple copies of the gene is a multiple, is typically noted as the gene symbol followed by the allele star number × N (*2 × N). If the protein is an enzyme involved in drug metabolism such as one of the CYP P450 enzymes, increased enzyme activity can result in accelerated metabolism and altered pharmacokinetics. Conversely, CNV can also lead to the deletion or skipping of a gene leaving an individual with less than 2 copies. Lacking one or both copies of a *CYP P450* gene can result in less to no enzyme being produced leading to poor drug metabolism.

Variation can also exist in the factors that regulate the expression of genes but do not alter the nucleotide base sequence. Epigenetics, meaning above or upon the gene, is the study of factors and processes within the cell or the environment that influence the expression or suppression of the genes mapped in the DNA sequence. In other words, epigenetic factors such as methylation and transcription factor proteins signal the static DNA blueprint to turn on and off.

An organization called the Pharmacogene Variation Consortium (PharmVar.org) has been formed to catalog and consistently name pharmacogene variants, especially alleles of the CYP P450 enzymes.^15^ As clinical genetic testing becomes widespread, it is important for researchers and providers to communicate PGx results in an accurate and standardized manner. As in the above example, variants of the CYP P450 enzymes are customarily named using star-allele nomenclature where the gene symbol is followed by an asterisk and a variant number. PharmVar will expand upon this convention and also serve as a repository for phenotypic information on pharmacogenes. Variant tables for the polymorphic *CYP2C9, CYP2C19*, and *CYP2D6* genes are posted in the PharmVar database and include the corresponding phenotypic enzyme activity level.^16^

## Clinically Actionable Pharmacogene Variation

Generally, a gene-drug association is considered clinically actionable if information about genetic testing is included in the FDA-approved labeling. Currently, multiple gene-drug associations that are clinically actionable for pharmacists involve the CYP P450 enzymes. Variation in the DNA nitrogenous base sequence can produce different versions (alleles) of the *CYP2C9, CYP2C19*, and *CYP2D6* genes.^17^ Several known gene variants in turn produce CYP P450 enzymes that differ in the extent of enzyme activity or amount produced, and therefore in their ability to metabolize drugs. Knowing the genotype or the 2 gene alleles a person inherits gives the pharmacist additional information to consider when selecting or dosing drugs. To illustrate several of these concepts, let us examine how *CYP2D6* gene variation relates to both the effectiveness and safety of the opioid analgesic drug codeine.

## *CYP2D6* Polymorphism

The CYP2D6 enzyme participates in the metabolism of approximately 25% of all drugs, including the bioactivation of codeine, tramadol, and several other pro-drugs. The *CYP2D6* gene located on chromosome 22 is highly polymorphic and over 90 known variants or alleles have been identified which encode for multiple forms of the enzyme.^18^ For many of these alleles, a relationship has been established between the version of the gene and the enzymatic activity of the CYP2D6 protein produced. For example, the CYP2D6*4 allele results from a SNP caused by the substitution of an adenine instead of a guanine (G>A), which shifts the code and produces a non-functioning enzyme. The *CYP2D6* gene is also subject to inheritable CNV, which can include both multiple copies of the gene or deletion of one or both copies. One study found that 12.6% of 30 000 patient samples tested contained 0, 1, or 3 or more copies of the *CYP2D6* gene.^19^

Codeine is essentially a pro-drug that needs the CYP2D6 enzyme to convert 5% to 10% of each dose to morphine, which has a 200 times greater affinity for the mu opioid receptor than does the parent compound.^20^ The balance between adequate analgesic effect and over-sedation is dependent upon an individual’s CYP2D6 activity. Based on genotype (the combination of inherited alleles), people can be placed into 1 of 4 phenotypic categories that predict their expected level of CYP2D6 enzyme activity. Variation in the *CYP2D6* gene leads to a spectrum of enzymatic activity that ranges from rapid conversion of codeine to morphine to an inability to metabolize codeine through this pathway. Table 1 shows some common *CYP2D6* alleles accompanied with the phenotypic enzyme activity level.^21^ When presented with a person’s genotype, the phenotype category is assigned based on the highest functioning *CYP2D6* allele. People categorized as Ultrarapid Metabolizers (UMs) possess multiple copies (more than 2) of active alleles and express greater amounts of the CYP2D6 enzyme and therefore rapidly convert codeine to morphine. An example genotype of an UM might consist of a *1 allele (wild-type) and the CNV *2 × N (*1/*2 × N) resulting in the production of greater than normal amounts of the CYP2D6 enzyme activity. Ultrarapid Metabolizers can experience high blood levels of morphine and if breastfeeding can pass excessive amounts to a nursing infant. At the opposite end of the spectrum, PMs possess 2 no function alleles that encode for CYP2D6 enzyme that is inactive or does not get produced at all. When given usual doses of codeine, PMs experience little analgesic effect because they do not convert codeine to morphine to an appreciable degree. Extensive Metabolizer (EM) and Intermediate Metabolizer (IM) phenotypes are often grouped together and are typically considered to have enzyme activity within the normal range and classified as Normal Metabolizers (NMs). An individual with a genotype of *1/*17, for example, would be classified as an NM because they possess at least 1 normal function allele. Intermediate Metabolizers possess a genotype consisting of 2 decreased function alleles or 1 decreased and 1 no function allele such as *9/*41 or *9/*3.

**Table 1.** Cytochrome P450 *CYP2D6* allelic variants and enzyme activity.

Phenotype: *CYP2D6* enzyme activity level	*CYP2D6* gene variant (CYP2D6*X)
No function (null) alleles	*3 *4 *5 *6 *7 *8 *11 *12 *13 *15 *18 *19 *20 *21 *31 *36 *38 *40 *42 *44 *47 *51 *56 *57 *60 *62 *68 *69 *92 *96 *99 *100 *101 *114
Decreased function alleles	*9 *10 *14 *17 *29 *41*49 *50 *54 *55 *59 *72 *84
Normal function alleles	*1 (wild type) *2 *27 *33 *34 *35 *39 *45 *46 *48 *53
Increased function alleles	Copy number variants *1 × N *2 × N *35 × 2
Uncertain/unknown function alleles	*22 *23 *24 *25 *26 *28 *30 *37 *43 *52 *58 *61 *63 *64 *65 *70 *71 *73 *74 *75 *81 *82 *83 *85 *86 *87 *88 *89 *90 *91 *93 *94 *95 *97 *98 *102 *103 *104 *105 *106 *107 *108 *109 *110 *111 *112 *113

Source: Adapted from Pharmacogene Variation Consortium (PharmVar).^21^

Once the *CYP2D6* genotype and metabolizer class is determined, the pharmacist must integrate this information into the drug treatment plan. Consulting the PharmGKB is a vital first step. PharmGKB.org provides a comprehensive and easily accessible online PGx resource developed and generously shared by experts at Stanford University. The mission of PharmGKB is to collect, curate, and disseminate knowledge about the impact of human genetic variation on drug response.^22^ It is an invaluable repository of evidence-based PGx knowledge that is organized and annotated for clinicians and researchers alike. In addition to the aforementioned list of VIPs, PharmGKB houses clinical practice guidelines and drug labeling information from the United States, Canada, Japan, and the Netherlands.

Navigating PharmGKB can be as simple as entering the name of the drug or gene into the search field and arriving at a monograph-type synopsis with a side bar section tool. Another efficient way to navigate is to select the category link titled *Dosing Guidelines* displayed on the home page and scrolling to the drug name such as codeine, for example. Displayed here is a side-by-side selection of the available vetted PGx clinical practice guidelines for codeine.^23^ Accessing the link in the first column displays an annotation of the Clinical Pharmacogenetics Implementation Consortium (CPIC) Guidelines concerning codeine and *CYP2D6*. The CPIC Guidelines are composed by an international expert panel with a mission to help clinicians use genetic testing to inform safe prescribing (https://cpicpgx.org/). The CPIC recommends using alternate analgesics for people categorized as UMs and PMs. For those categorized as NMs (IMs and EMs), CPIC advises following the FDA labeling recommendations which contraindicate the use of codeine in children less than 18 years old and in breastfeeding women. Next, guidelines from the Royal Dutch Association for the Advancement of Pharmacy—Pharmacogenetics Working Group (DPWG) are posted for comparison. Notice that DPWG recommendations differ from CPIC by advising that IMs avoid codeine use as well as UMs and PMs. The last link leads to the Canadian Pharmacogenomics Network for Drug Safety (CPNDS) guidelines that match CPIC recommendations on codeine use and *CYP2D6* genotype.

## Predicting the Risk for Adverse Drug Reactions

One of the most promising applications of PGx information is the ability to predict an individual patient’s risk for developing certain drug side effects. Approximately 15% of adverse drug reactions (ADRs) are Type B or idiosyncratic reactions, meaning they are not related to the expected pharmacology, pharmacokinetics, or systemic concentration of a medication.^24^ Often these are immune-mediated, hypersensitivity reactions that in some cases have been associated with genetic risk factors. Several strong associations between variants of the Human Leukocyte Antigen (*HLA*) gene complex and the occurrence of severe cutaneous reactions have been established for a range of drugs.^25^ For this reason, PharmGKB designates the Human Leukocyte Antigen B (*HLA-B*) gene as a VIP and provides links to CPIC guidelines on its website, which address *HLA* gene testing and safe use recommendations for abacavir, allopurinol, phenytoin, oxcarbazepine, and carbamazepine.^26^

The *HLA-B* gene is part of a complex of genes located on chromosome 6 that encode for cell surface proteins involved in presenting antigens to the immune system. Specific allelic variants of *HLA-B* have been associated with the development of severe cutaneous ADRs following exposure to carbamazepine typically occurring early in therapy. Known as Stevens-Johnson Syndrome (SJS) and Toxic Epidermal Necrolysis (TEN), these maladies can result in blistering and sloughing of the skin and mucous membranes that can proceed to liver and other organ failure.^27^ Stevens-Johnson Syndrome and TEN are really a continuum of severity of the same adverse reaction. Stevens-Johnson Syndrome is fatal in 10% of patients, whereas 50% of those who progress to TEN die.

Possessing 1 copy of the HLA-B*1502 allele places a person receiving carbamazepine at a greater risk for experiencing these rare but sometimes fatal delayed hypersensitivity reactions. For this reason, the FDA mandates as a Boxed Warning targeted genetic testing for the HLA-B*1502 allele before initiation of carbamazepine in ethnic groups more likely to possess this allelic variant and to avoid use in carriers. These groups include people of Han Chinese, Thai, Malaysian, Indonesian, Filipino, and South Indian ethnicity who have as much as 10 times the risk for experiencing hypersensitivity reactions than do mainly Caucasian populations. A review of the FDA Table of Pharmacogenomic Biomarkers in Drug Labeling identifies the location within the drug labeling of any recommendation regarding genetic testing.^28^ Notice for carbamazepine that the FDA also suggests but does not require testing for another *HLA* complex gene variant, the HLA-A*3101 allele, which is associated with an increased risk of drug reaction with eosinophilia and systemic symptoms (DRESS) and maculopapular exanthema (MPE) as well as SJS/TEN. This differs from CPIC guidelines for carbamazepine and *HLA-A* and *HLA-B* gene variants, which recommend that testing for both HLA-B*1502 and HLA-A*1301 be conducted for all carbamazepine-naïve patients in high-risk ethnic groups.^29^ For clearly actionable and FDA required genetic testing such as for new-starts on carbamazepine in specific ethnic populations, pharmacists can play an important role within their practice institutions by inquiring and conducting reviews to determine whether appropriate genetic testing is being done.

## Genetic Sequencing

The increasing accessibility and affordability of genetic testing is driving the translation of genomic medicine into clinical practice. Improvements in DNA sequencing technologies have dramatically reduced both the cost and the time it takes to accomplish whole genome sequencing (WGS) and whole exome sequencing (WES), which selectively maps the protein-coding or exon regions of the genes. The HGP spent in excess of $500 million and took over 10 years to sequence the first single reference human genome.^30^ By comparison, in 2018, WGS conducted in standard clinical practice can deliver results in 2 to 8 weeks at a cost of less than $1000. Testing for targeted gene variants can be accomplished in a matter of days at a cost of a few hundred dollars.

In 1977, Frederick Sanger developed the original base sequencing technique that involved cleaving 1 strand of the DNA molecule into millions of pieces, copying or amplifying the fragments, and painstakingly identifying the terminal nitrogenous base in each fragment by tagging it with a radioactive or fluorescent complementary base. The HGP used an updated version of the Sanger Method that automated the process and allowed multiple fragments to be tested at one time. Today’s technologies called next-generation or high-throughput sequencing can sequence millions of DNA fragments simultaneously and process DNA from multiple individuals at the same time.^31^ An entire genetic testing industry has arisen devoted to exploring new technologies and marketing services not only to health care providers but directly to consumers.^32^

Perhaps because of the success and public receptivity of ancestry genetic testing, personal medical testing is being marketed directly to the public with at-home DNA sample collection. Many companies offer clinical-grade genetic testing after delivery of a saliva sample and some but not all require a physician’s order. Typically, a package of targeted genes intended to screen for an individual’s risk for developing specific diseases are characterized. Several companies offer to test for gene variants predictive of cancers or inherited cardiovascular diseases such as cardiomyopathy. A few companies specifically offer PGx testing with 1 testing for 50 pharmacogenes. The direct-to-consumer marketing of genetic testing places the results into the consumer’s hands, and while most companies offer access to genetic counselors or help lines, people are often directed to discuss testing results with their health care providers. Pharmacists may be called upon to evaluate a person’s drug regimen and tailor it based on PGx test results. Reports usually include a clinical interpretation to help the recipient understand the implications of the test. If presented with a report from a patient, accessing the company’s website to obtain more information about which genes are tested and access clinical decision support tools may be helpful. Certainly, consulting PharmGKB and CPIC guidelines will be useful.

## Conclusions

Pharmacists are the ideal providers to orchestrate the translation of PGx gene-drug associations into clinical practice and improve patient care. Knowing the genetic information of our patients adds complexity to treatment options and gives us new attributes to consider when personalizing the selection, dosing, and monitoring of drug therapy. Discovering and getting involved with committees focused on genomic medicine at your institution is important for establishing our eventual role early-on. Consider adding PGx subcommittees to established pharmacy committees such as Pharmacy and Therapeutics and Medication Safety.

Hopefully, this review has inspired you to embrace this emerging body of knowledge and continue to learn about new discoveries in genomic medicine. As PGx driven drug therapy advances, in medical science lead to a paradigm shift in drug discovery and treatment, it is both necessary and professionally fulfilling to keep current and practice ready.

## Supplemental Material

Self_Assessment_Test_Questions_Pharmacogenomics_-_The_Time_is_Now_xyz268532ab00280 – Supplemental material for Basic Concepts in Genetics and Pharmacogenomics for Pharmacists Click here for additional data file. Supplemental material, Self_Assessment_Test_Questions_Pharmacogenomics_-_The_Time_is_Now_xyz268532ab00280 for Basic Concepts in Genetics and Pharmacogenomics for Pharmacists by Kathleen B Orrico in Drug Target Insights
