# Supplementary material for: Basic Concepts in Genetics and Pharmacogenomics for Pharmacists
Source: Drug Target Insights. 2019 Dec 3;13:1177392819886875. doi: 10.1177/1177392819886875 (PMC6891005; doi:10.1177/1177392819886875)
Supplement: Self_Assessment_Test_Questions_Pharmacogenomics_-_The_Time_is_Now_xyz268532ab00280 – Supplemental material for Basic Concepts in Genetics and Pharmacogenomics for Pharmacists [file Self_Assessment_Test_Questions_Pharmacogenomics_-_The_Time_is_Now_xyz268532ab00280.pdf]

Self-Assessment Questions & Key – Pharmacogenomics – The Time is Now 2/7/19

1. Which of the following statements best summarizes the main goal of the Human Genome Project.
    a. To identify the amino acid sequence of every protein molecule human beings produce.
    b. To identify the sequence of nucleotide base-pairs in nuclear human deoxyribonucleic acid (DNA).
    c. To lay claim to DNA sequencing information for the United States.
    d. To determine the structure and conformation of the 23 human chromosomes.

2. The structure of deoxyribonucleic acid (DNA) includes the following:
    a. Four amino acids that spell out the code of life.
    b. The pyrimidines adenine and thymine and the purines guanine and cytosine.
    c. Nucleotide subunits consisting of a nitrogenous base and the sugar ribose linked by an acidic phosphate group.
    d. The purines adenine and guanine and the pyrimidines cytosine and uracil.

3. Which phrase below best describes the term epigenetics?
    a. The study of gene variants that are known to produce differences in drug response between individuals.
    b. The science that relates factors within the cellular environment to gene expression and suppression.
    c. A domain within molecular biology that seeks to determine the structure and function of the DNA of an organism.
    d. The study of gene-editing.

4. Which single stranded DNA sequence listed below is complementary to the sequence ACCATGGA in the <u>double stranded</u> sequence?
    a. TGGTAGTC
    b. TGCGCTCA
    c. UGGUCCU
    d. TGGTACCT

5. Which of the following examples is a phenotype?
    a. The substitution of an Adenine for a Cytosine
    b. Blue eye color
    c. HLA-B *1502
    d. Multiple copies of the CYP2D6 gene

6. What is the best source for determining if genetic testing is required for a given drug?
    a. The FDA Table of Pharmacogenomic Biomarkers in Drug Labeling
    b. The FDA MedWatch
    c. Bookshelf at National Center for Biotechnology Information – NCBI
    d. The National Library of Medicine's Genetics Home Reference

7. Identify the main driver of the translation of pharmacogenomics (PGx) into clinical practice.
    a. **Improvements in genetic testing technology.**
    b. The changing attitudes of pharmacists and other providers.
    c. FDA Clinical Practice Guidelines.
    d. PGx offers the potential to reduce health care cost.

8. The length of this portion of a chromosome may be related to the age of a cell.
   a. An allele
   b. An intron
   c. An exon
   d. A telomere

9. Use Table 1. to determine which of the groups below contains CYP2D6 genotypes from Intermediate Metabolizers.
   a. *3/*9, *9/*17, *3/*10A
   b. *3/*4, *7/*31, *40/*42
   c. *1/*1, *2A/*17, *27/*33
   d. *1/*1 x N, *1 x N/*2A

10. What is the best source for retrieving comprehensive and curated PGx information?
   a. The FDA Table of Pharmacogenomic Biomarkers in Drug Labeling
   b. The FDA MedWatch
   c. The Pharmacogenomics Knowledge Base or PharmGKB
   d. The National Library of Medicine's Genetics Home Reference

KEY - Self-Assessment Questions –Pharmacogenomics – The Basics for Pharmacists 9/2018 – Orrico KB

1. Answer: b is correct. The goal of the HGP was to identify and correctly sequence sequence the 3 billion DNA base pairs that make up the human genome. Selection a. is a description of the Human Proteome Project which followed HGP with a goal to identify and map proteins coded by genes. Selection c. Incorrect because the HGP was a worldwide effort. Selection d. A better understanding of chromosomes did result from the HGP but was not the main intent.
2. Answer: c is correct. Selection a. is incorrect because DNA contains 4 distinct nitrogenous bases. Proteins contain amino acids. Selection b is incorrect because adenine and guanine are purines and thymine and cytosine are the pyrimidines contained in DNA. Selection d is incorrect because the purines adenine and guanine and the pyrimidines cytosine and uracil are the nitrogenous bases contained in RNA ribonucleic acid.
3. Answer: b. is correct. Selection a. is the definition of Pharmacogenomics. Selection c. is the definition of Genomics. Selection d. Gene-editing is an emerging technique directed at altering the DNA.
4. Answer: d. is the complementary strand. Adenine always pairs with Thymine and Cytosine always pairs with Guanine in the structure of DNA.
5. Answer b. is correct because a phenotype is a description of the observable physical characteristic such as eye color that results from the expression of genes. Selection a. describes a SNP or single nucleotide polymorphism variation. Selection c. is an allele or version of a gene. Selection d. is a CNV or copy number variation.
6. Answer: a. Drug labeling will contain the exact requirement of the FDA. If PharmGKB was an option it also would be a great source of this information in the 'Drug Labeling' section. The other references may contain information regarding the value of testing but a. is the best choice amongst this group.
7. Answer: a. The continued reduction in the cost of testing and the turn-around time of newer sequencing technologies is the main reason PGx testing is being translated into practice. Selection b.,c.,d. all have bearing but improvements in technology is the main reason.
8. Answer: d. Telomeres are sequences of bases at the ends of the chromosomes that participate in DNA repair and are associated with the length of the cell's lifespan. An allele is a gene variant. Introns are the

sections of the gene sequence within the gene that are regulatory and the exon portions contain the protein-coding bases.

9. Answer: a. All genotypes in this group are classified as CYP2D6 Intermediate Metabolizers because they each contain at least one decreased function allele as the highest activity allele. Selection b contains Poor Metabolizers and are groupings of no function alleles. Selection c. displays Extensive Metabolizers that possess at least one normal activity allele as the highest functioning. d. contains Ultrarapid Metabolizers.
10. Answer: c. While the other sources listed do contain helpful PGx information, PharmGKB is the most comprehensive up-to-date choice as well as a means to accessing vetted clinical practice guidelines.
